# Atypical Hemolytic Uremic Syndrome: Differential Diagnosis from TTP/HUS and Management

**DOI:** 10.4274/tjh.2013.0374

**Published:** 2014-09-05

**Authors:** Mustafa N. Yenerel

**Affiliations:** 1 İstanbul University İstanbul Faculty of Medicine, Department of Internal Medicine, Division of Hematology, İstanbul, Turkey

**Keywords:** Atypical hemolytic uremic syndrome (aHUS), Thrombotic thrombocytopenic purpura (TTP), eculizumab, TTP/HUS, Thrombotic microangiopathy (TMA), ADAMTS13

## Abstract

Atypical hemolytic uremic syndrome (aHUS) is a rare form of thrombotic microangiopathy (TMA). It has an unfavorable outcome with death rates as high as 25% during the acute phase and up to 50% of cases progressing to end-stage renal failure. Uncontrolled complement activation through the alternative pathway is thought to be the main underlying pathopysiology of aHUS and corresponds to all the deleterious findings of the disease. Thrombotic thrombocytopenic purpura (TTP) and Shiga toxin-associated HUS are the 2 other important TMA diseases. Although differentiating HUS from TTP is relatively easy in children with a preceding diarrheal illness or invasive S. pneumoniae, differentiating aHUS from TTP or other microangiopathic disorders can present a major diagnostic challenge in adults. ADAMTS13 analysis is currently the most informative diagnostic test for differentiating TTP, congenital TTP, and aHUS. Today empiric plasma therapy still is recommended by expert opinion to be used as early as possible in any patient with symptoms of aHUS. The overall treatment goal remains restoration of a physiological balance between activation and control of the alternative complement pathway. So it is a reasonable approach to block the terminal complement complex with eculizumab in order to prevent further organ injury and increase the likelihood organ recovery. Persistence of hemolysis or lack of improvement of renal function after 3-5 daily plasmaphereses have to be regarded as the major criteria for uncontrolled TMA even if platelet count has normalized and as an indication to switch the treatment to eculizumab. Eculizumab has changed the future perspectives of patients with aHUS and both the FDA and the EMA have approved it as life-long treatment. However, there are still some unresolved issues about the follow-up such as the optimal duration of eculizumab treatment and whether it can be stopped or how to stop the therapy.

## OZET

Atipik hemolitik üremik sendrom (aHÜS) trombotik mikroanjiopatilerin nadir görülen bir şeklidir. Kötü seyirli bir sendrom kabul edilen aHÜS olgularında özellikle akut dönemlerinde %25’e varan oranlarda ölüm riski mevcuttur ve yine olguların %50’ sinde son dönem böbrek yetersizliği ile sonuçlanır. Komplemanın alternatif yolağının kontrolsüz aktivasyonu sonucu oluştuğuna inanılan aHÜS olgularında tüm klinik bulgulardan da yine bu kontrolsüz kompleman aktivasyonu sorumlu tutulmaktadır. Trombotik trombositopenik purpura (TTP) ve Shiga toksinle ilişkili HÜS diğer iki önemli TMA nedenidir. TTP ve HÜS ayırımı özellikle çocuklarda hastalığın hemen öncesinde saptanan diyare ya da invazif pnömoni varlığı sayesinde kolaylıkla yapılabilmektedir. Fakat erişkinde aHÜS olgularını TTP olgularından ya da diğer TMA nedenlerinden ayırmak zordur. ADAMTS13 analizi günümüzde TTP, konjenital TTP ve aHÜS olgularının ayırımında kullanılan en önemli inceleme yöntemidir. Günümüzde aHÜS bulgularıyla başvuran hastalarda acil olarak plazma tedavisine başlanması hala uzman görüşü olarak tavsiye edilen bir tedavi seçeneğidir. Asıl tedavi beklentisi ise alternatif kompleman yolağının aktivasyonu ve kontrolü arasındaki fizyolojik dengenin sağlanmasıdır. Bu nedenle özellikle daha fazla organ hasarı gelişmesini önlemek ve oluşan hasarın düzeltilmesini sağlayabilmek için en makul tedavi seçeneği eculizumab ile terminal kompleman kompleksinin oluşmasının engellenmesidir. Kontrolsüz TMA olgularında özellikle üç ya da beş günlük plazmaferez tedavisiyle hemolizin kontrol altına alınamaması ve böbrek foksiyonlarında düzelme sağlanamaması trombositler düzelse bile eculizumab tedavisine geçilmesi için major kriter kabul edilmektedir. Eculizumab hem FDA hem de EMA tarafından aHÜS olgularının tedavisinde hayat boyu kullanımı için onaylanmış bir tedavidir ve günümüzde aHÜS olgularının seyrini değiştirmiştir. Fakat eculizumab tedavisinin aHÜS tedavisindeki optimum süresinin ne olduğu, takip sırasında ilacın kesilip kesilemeyeceği, ya da nasıl kesileceği gibi konular henüz açıklığa kavuşmamıştır. 

## INTRODUCTION

Hemolytic uremic syndrome (HUS) is a rare and severe thrombotic microangiopathy (TMA) characterized by the triad of microangiopathic hemolytic anemia, thrombocytopenia, and renal impairment. The disorder occurs most frequently in children under the age of 5 years, with an overall incidence of 1 to 2 cases per 100,000. The disease is generally caused by infectious agents and usually has a favorable outcome. Approximately half of the patients require dialysis during acute episodes, but renal function recovers in most of them [1,2]. A history of diarrhea is essential to define the diagnosis of postinfectious HUS and is generally seen 2 weeks before the episode [3]. The enterohemorrhagic Escherichia coli serotypes producing Shiga toxin are the most common infectious agents causing HUS. Shigella is the second important infectious agent that can cause HUS with the deleterious effects of Shiga toxins. Streptococcus pneumoniae is another important infectious agent that can provoke more aggressive clinical forms [4,5,6,7]. Canadian pediatricians compared Streptococcus pneumoniae-related HUS cases with Shiga toxin-associated HUS and found that Streptococcus pneumoniae-related HUS patients were more likely to require dialysis and had a longer duration of hospitalization [8]. E. coli-associated HUS often occurs in clusters or outbreaks. In the absence of outbreaks, physicians are more likely to encounter atypical HUS (aHUS) than Shiga toxin-associated HUS.

Approximately 5%-10% of HUS cases are classified as aHUS because they are not caused by Shiga toxin-producing bacteria or streptococci; these cases comprise a heterogeneous group of patients. The clinical outcome is unfavorable in this group, with death rates as high as 25% during the acute phase and up to 50% of cases progressing to end-stage renal failure (ESRF) [9,10]. This article reviews current concepts about the pathophysiology of aHUS and differential diagnosis from thrombotic thrombocytopenic purpura TTP/HUS and management.

**Epidemiology**

aHUS is considered to be an extremely rare disease. Although there are limited data available in the literature about the incidence and prevalence of this entity, the estimated incidence rate was given as 1-2 cases per million annually in the United States [7]. In the European HUS registry, 167 patients were identified and the calculated prevalence of aHUS/recurrent HUS was reported as 3.3 per million-child population (<18 years) [11]. The age distribution is different and the prevalence in adults is expected to be lower, but clear data are not yet available. aHUS represents 5%-10% of HUS cases in children, but the majority of HUS is seen in adults. The incidence of complement-aHUS is not known precisely. However, more than 1000 aHUS patients investigated for complement abnormalities have been reported [12].

**Pathophysiology of Thrombotic Microangiopathies**

TMA is a pathological process characterized by thickening of arterioles and capillaries, endothelial swelling and detachment, subendothelial accumulation of proteins and cell debris, and fibrin and platelet thrombi obstruction of vessel lumina [13]. TMA predominantly affects the renal microvasculature, although the brain, heart, lungs, and gastrointestinal tract may also be involved, ultimately leading to organ dysfunction [14,15,16,17,18]. TMA may result from 4 types of lesions: von Willebrand factor (VWF)-platelet thrombi with no or minimal microangiopathy; fibrin-platelet thrombi, as exemplified by disseminated intravascular coagulopathy (DIC); inflammatory or proliferative microangiopathy accompanied with variable fibrin thrombi; or intravascular clusters of cancer cells [19].

TTP was originally defined pathologically as a systemic disease with widespread VWF-platelet thrombi in the arterioles and capillaries of multiple organs [20,21]. Advances in recent years have demonstrated that VWF-platelet thrombi result from a disintegrin and metalloproteinase with a thrombospondin type 1 motif, member 13 (ADAMTS13) deficiency. TTP is now defined as a thrombotic disorder resulting from severe ADAMTS13 deficiency. Autoimmune inhibitors against ADAMTS13 account for most of the cases, known as acquired TTP [22]. Genetic mutations are also found in a small number of patients with ADAMTS13 deficiency [19,23]. In Shiga-like toxin-producing E. coli HUS, the toxin triggers endothelial complement deposition through the upregulation of P-selectin and possibly interferes with the activity of complement regulatory molecules [24]. Evidence shows that Shiga toxins might directly contribute to complement activation as documented by C3 deposition on microvascular endothelial cell lines exposed to Shiga toxin and then perfused with human serum [25]. 

Complement activation products have been detected in the serum and plasma of HUS patients, and an in vitro study could show that Shiga toxin not only damages the kidneys directly but also indirectly via a complement, in 2 ways. First, it activates the complement, and second, it delays the functions of its control protein factor H on the cell surface, both known to damage the kidney [26,27]. Today, TMAs are classified into 4 groups. Group I includes TTP with severe ADAMTS13 deficiency due to autoimmune inhibitors or genetic mutations. Group II includes aHUS with defective complement regulation due to genetic mutations or autoantibodies of the activators or regulators of the complement pathway. Group III includes TMA via other mechanisms such as toxin-associated or drug-related HUS, and Group IV includes other types of pathology such as fibrin platelet thrombosis (e.g., DIC, HELLP syndrome, catastrophic antiphospholipid syndrome, heparin-induced thrombocytopenia, or paroxysmal nocturnal hemoglobinuria) or malignancy- and vasculitis-associated thrombosis. Tsai reported that comorbid conditions such as infections, inflammation, surgery, trauma, pregnancy, intravenous contrast agents, and pancreatitis may trigger acute presentation in patients with preexisting TTP or aHUS, either by promoting the VWF-platelet interaction or by activating the complement system [28]. Tsai further subclassified groups I and II in terms of having one of these comorbidities or not. TTP, Shiga toxin-associated HUS, and aHUS are 3 main TMA diseases that have significant clinical overlap with patients presenting with similar signs and symptoms [13,29]. In aHUS, the predominant pathological abnormality is found in the renal arterioles and interlobular arteries. There is widespread endothelial swelling with retraction leading to exposure of the basement membrane. The vessel lumens are occluded by red cells and platelet fibrin thrombi. This preglomerular picture differs from Shiga toxin-associated HUS, where the pathology predominantly affects the glomerular capillaries [30,31].

Differentiating TTP from aHUS can present a major diagnostic challenge. TTP is characteristically diagnosed when neurological features predominate, although HUS is suspected when renal failure predominates. Because of these overlapping and changing presentations, some investigators viewed TTP and HUS as one disease with a spectrum of organ involvement. Historically, the term TTP/HUS was widely used for situations in which the clinical symptoms did not fit clearly into either category, and it is difficult to make a differential diagnosis without ADAMTS13 assays. ADAMTS13 assays are now available for most clinical practices, and they are crucial for the diagnosis of TTP and its differential diagnosis from aHUS.

During the last 2 decades, 4 regulatory proteins of the complement alternative pathway were shown to have a role in the pathogenesis of aHUS. Mutations in factor H and membrane cofactor protein (MCP) were the first mutations that helped to establish that aHUS is a disease of complement dysregulation. More than 50 different mutations in complement factor H (CFH), a plasma protein that inhibits the activation of the alternative pathway of the complement, have been described in aHUS cases. The majority of them are heterozygous and cause either single amino acid substitutions or premature translation interruption within the protein C-terminus, where binding sites for C3b/3d and heparin have been mapped [32]. If patients have defects in MCP or any other complement inhibitory proteins, even if heterozygous, they will probably be at increased risk for severe tissue damage because they cannot appropriately regulate C3 activation and amplification. The identification of factor H and MCP mutations has substantially enhanced the understanding of the molecular pathogenesis of atypical HUS [33]. During analysis of aHUS patients more mutations were found affecting other regulatory proteins, such as complement factor I (CFI) and thrombomodulin (THBD) and 2 proteins of the C3 convertase, C3 and factor B (CFB). All these findings proved that uncontrolled complement activation through the alternative pathway is the main underlying pathophysiology of aHUS [34,35,36,37,38,39,40,41,42,43,44,45,46]. Development of autoantibodies directed especially against CFH was identified as another form of aHUS in 2005 [46]. It has also been shown that presence of anti-CFH autoantibodies leads to an acquired and transient CFH deficiency [47,48].

A genetic mutation in complement regulatory proteins or by autoantibodies to CFH has been found as an explanation for constitutive complement activation in 50%-60% of cases of aHUS [31]. However, there are still 40%-50% of patients in whom a mutation cannot be demonstrated. Thus, the ultimate diagnosis of aHUS does not require a formal demonstration of its underlying genetic cause. 

**Clinical Findings of aHUS**

The onset of aHUS is generally sudden. Most patients have the complete triad of hemolytic uremic syndrome with anemia, thrombocytopenia, and renal failure, with or without anuria or reduced urine volume, and proteinuria if diuresis is maintained. Microangiopathic hemolysis is confirmed by the presence of schistocytes, low haptoglobin, and high lactate dehydrogenase levels. Patients usually complain of fatigue and general illness. Extrarenal manifestations are observed in 20% of patients and most of them (10%) are related to central nervous system involvement (CNS) [12]. CNS involvement is usually manifested by irritability, drowsiness, seizures, diplopia, cortical blindness, hemiparesis/hemiplegia, stupor, or coma [12].

If diagnosis is delayed, life-threatening hyperkalemia, acidosis, and volume overload with arterial hypertension and hyponatremia may be observed. Arterial hypertension is frequent and often severe, due both to volume overload in the case of oliguria/anuria and to hyperreninemia secondary to renal TMA. Cardiac failure or neurological complications (seizures) due to hypertension are possible. Myocardial infarction due to cardiac microangiopathy has been reported in 3% of patients [16,49]. Distal ischemic gangrene can also occur [50]. Half of children and the majority of adults need dialysis at admission [12]. Multiorgan failure due to diffuse TMA is present in 5% of patients [17,49]. 

**Differential Diagnosis**

If a patient comes to the clinic with microangiopathic hemolytic anemia, thrombocytopenia, and renal failure preceded by abdominal pain and diarrhea, the diagnosis has usually been Shiga toxin-associated HUS. This type of presentation accounts for 90% of HUS cases in children. Bloody diarrhea and abdominal pain are usually manifestations of hemorrhagic enterocolitis caused by Shiga toxin-producing bacteria, most commonly Escherichia coli O157:H7 [3]. Patients with Shiga toxin-associated HUS have been described that do not have obvious diarrhea prodromes. On the other hand, patients with aHUS commonly present with abdominal pain and diarrhea, which may begin days to weeks before the patients seek medical care, thus giving rise to the impression of diarrhea-associated HUS. Diarrhea, with or without blood, is found in approximately 30% of patients with aHUS at the disease onset because of the gut involvement [17,51]. For this reason, categorization of HUS according to clinical presentation as diarrhea-positive or diarrhea-negative is inappropriate [52].

If a patient presents with a diarrheal prodrome, Shiga toxin-associated HUS can easily be differentiated with the presence of Shiga toxin in the stools (done by the Vero cell assay) and/or serum antibodies against Shiga toxin (by enzyme-linked immunosorbent assay) and/or antilipopolysaccharide antibodies against the most common serotypes in the country in question. Another infectious form of HUS occurs with T-antigen activation in association with pneumococcal sepsis and this can also easily be differentiated from aHUS.

Although differentiating HUS from TTP is relatively easy in children with a preceding diarrheal illness or invasive S. pneumoniae, differentiating aHUS from TTP or other microangiopathic disorders can present a major diagnostic challenge in adults. Clinical presentation is also more confusing in adults and it is difficult to make a differential diagnosis without ADAMTS13 assays.

ADAMTS13 assays are now available for most clinical practices. In contrast, the mutation analysis tests for the diagnosis of aHUS are performed primarily in research laboratories. Furthermore, the molecular defects of aHUS remain unknown in many cases. Therefore, for patients presenting with thrombocytopenia and microangiopathic hemolysis, ADAMTS13 analysis is currently the most informative diagnostic test for differentiating TTP, congenital TTP, and aHUS. 

Plasmapheresis should be started as soon as possible in any patient presenting with microangiopathic hemolytic anemia and thrombocytopenia without waiting for the results of other diagnostic investigations.

It also should not be forgotten to take plasma samples and store them at -20 °C for the analysis of ADAMTS13 activity and inhibitor levels before any plasma therapy. It usually takes less than 1 week in Turkey to get the test results. During that time, further investigations should also be done to exclude other possible causes of TMA, such as Shiga toxin-associated HUS, systemic lupus erythematosus, antiphospholipid syndrome, infections, malignancies, endothelial-insulting drugs, and chemotherapies [53,54]. Acute TTP can easily be diagnosed if we find the plasma ADAMTS13 activity level to be less than 10% (or 5%, depending on the assays). The deficiency is caused by either inhibitors of ADAMTS13 or mutations of the ADAMTS13 gene. If there is a high inhibitor titer in a patient with a low ADAMTS13 level, then the diagnosis is acquired TTP. Inhibitors are detectable in 80%-90% of patients with acquired TTP using the conventional 1:1 mixing study performed in most clinical labs. Therefore, negative inhibitor results do not exclude the diagnosis of acquired TTP. Mutation analysis, family investigation, and/or serial ADAMTS13 assays are often needed to determine whether a patient has congenital or acquired TTP. Measurements of ADAMTS13 levels may also be quite informative even in the remission state of TTP, and an ADAMTS13 level below 5% (or 10%) is associated with high risk of relapse in the near future. Plasma ADAMTS13 antigen, activity, and inhibitor levels are all normal or moderately decreased in aHUS patients. Even more than 10% ADAMTS13 activity in a patient suspected of TTP/HUS is important for the diagnosis of aHUS. However, there are different test designs developed for the analysis of ADAMTS13 activity and the assays are affected differently by a variety of conditions, such as plasma bilirubin, hemoglobin, or VWF levels. Interpretation of ADAMTS13 assay results requires correlation with the patient’s clinical status. Therefore, it is better to do these assays in reference laboratories [55]. Anti-CFH autoantibodies represent a significant etiology of aHUS, mainly in preadolescent children, but they may also be present in adults [46]. Therefore, screening of anti-CFH autoantibodies at the onset of the disease is also recommended, if possible.

**Management of aHUS**

aHUS has an unfavorable outcome, with death rates as high as 25% during the acute phase and up to 50% of cases progressing to end-stage renal failure. It is confirmed that progress in intensive care and dialysis opportunity has contributed to the decrease of mortality. For this reason, all patients suspected of having aHUS should be transferred to a specialized center that has dialysis and plasmapheresis facilities.

Until recently, there have been no specific therapies for aHUS; plasma therapy remained the first-line treatment of aHUS in all guidelines published before 2010 based on expert opinions rather than clinical trials [56,57,58]. Plasmapheresis can replace deficient proteins and remove antibodies against complement regulatory proteins, such as anti-CFH antibodies. It would be especially sufficient in patients with defective complement regulatory proteins such as CFH. However, patient outcomes were still reported as poor with this syndrome if it was treated solely with plasma therapy [17,49,59].

According to the Italian registry, plasma therapy was found to be effective in 63% of patients, but only 5% had complete recovery and evolution to death or end-stage renal disease was reported as 37% with this approach [49].

There are also some observations from case studies, especially in children, that early intensive plasma therapy can reverse aHUS and that long-term plasma therapy can prevent relapses and evolution to ESRF in CFH-mutated patients [58,60]. Although most of them had relapses during infections and were treated by intensification of plasma therapy, most patients who received plasma therapy only during acute episodes died or were in ESRF within less than 1 year [58,60,61,62,63].

Case studies also showed that plasma therapy responses change with complement mutations. For example, it was reported that only 25% of patients with CFI mutation had a response and 75% progressed to death or ESRF in the Italian registry [49]. Again, all of the 5 CFI-mutated patients had complete or partial remission in the acute phase of the disease, but all had relapses and all except 1 developed ESRF within a few weeks or months [12,44,64].

MCP is a transmembrane protein. MCP mutations account for 15% of the aHUS cases; because it is a transmembrane protein, we do not expect any beneficial effects with plasmapheresis. At least 90% of patients undergo remission from acute episodes, whether or not they receive plasma therapy [17,40,49]. Long-term plasma therapy also does not seem to be effective in those patients [65]. Plasma therapies can also provide some degree of help in C3, CFB, or THBD mutations. It has been reported that 88% of THBD-mutated patients had a response with plasma therapy, but 43% of them progressed to death and 13% of them progressed to ESRF [49,65]. Remission has been achieved in 2 and 3 patients with C3 and CFB mutations, respectively [66,67,68].

Plasmapheresis and the removal of the antibodies is the first-line treatment in patients with anti-CFH antibodies. Immunosuppressive treatment with steroids, intravenous cyclophosphamide, mycophenolate mofetil, azathioprine, or anti-CD20 should also be used during the follow-up period. However, there is still no standardized protocol for the duration or type of immunosuppressive therapy in patients with anti-CFH antibodies [12,18,69,70,71,72]. 

**Transplantation**

Any aHUS patient who has ESRF is theoretically a candidate for renal transplantation. However, the clinical outcome of renal transplantation in patients with aHUS is discouraging. Patients with aHUS are more prone to develop acute rejections, which also affects graft survival. Approximately half of the patient groups with aHUS will develop recurrent disease and graft loss [73].

The recurrence risk of aHUS after renal transplantation was found to be less than 1% in typical HUS patients. However, the recurrence risk increases up to 60% in aHUS patients [74]. Eculizumab therapy is expected to shift the paradigm [28]. With eculizumab started preoperatively and continued postoperatively, preliminary experience suggests that excessive morbidity, mortality, and kidney graft failure may be prevented [75,76].

Although there are no clinical predictors of outcome, knowledge of the underlying genetic defect is helpful in predicting prognosis [77]. The recurrence risk in patients with a CFH mutation is 75%-90%; for patients with a CFI mutation, it is 45%-80%, and in the case of a C3 mutation, the risk of an aHUS recurrence is 40%-70% [77,78]. Recurrences have been seen in patients with CFB and thrombomodulin mutations, as well. On the other hand, patients with a mutation in the gene encoding the membrane-bound MCP have a low risk of developing a disease recurrence in the graft [13]. MCP is cell membrane-bound and highly expressed in the kidney; kidney transplants, then, would be expected to halt the disease process [4]. Combined liver-kidney transplantation has been attempted for patients with CFH and CFI mutations to address the abnormal protein synthesis in the liver and its downstream effect on the kidney. Simultaneous liver-kidney transplantation with prophylactic use of plasma therapy has been successful in patients with CFH mutations [60]. However, liver-kidney transplantation is associated with a higher mortality rate than kidney transplantation alone [79]. In the absence of a noted mutation comprising a sizable fraction of patients with aHUS, liver-kidney transplantation should be avoided [13,80].

**Complement Inhibitor Therapy**

Eculizumab is a humanized monoclonal antibody that binds to complement C5 protein and prevents the formation of the terminal complement complex, also known as the membrane attack complex (MAC). This agent has been approved for paroxysmal nocturnal hemoglobinuria and was approved by the US Food and Drug Administration (FDA) for use in aHUS on 23 September 2011 [81]. In aHUS, uncontrolled activation of the alternative complement pathway corresponds to all the deleterious findings of the disease. The overall treatment goal remains restoration of a physiological balance between activation and control of the alternative complement pathway. Thus, it is a reasonable approach to block the terminal complement complex with eculizumab in order to prevent further organ injury and increase the likelihood of organ recovery.

The first case report using eculizumab as a therapeutic approach in aHUS was reported in 2009. Nurnberg et al. reported that an 18-month-old boy with a plasma-resistant congenital form of the disease achieved remission after the initiation of treatment with eculizumab [82]. The second important observation was the resolution of hemolysis and improvement of the transplant function after receiving eculizumab in a 30-year-old woman with a CFH mutation who had a recurrence of the hemolytic-uremic syndrome in a kidney graft [83].

Since these first reports, many case presentations have followed, demonstrating that good clinical responses have been observed when using eculizumab in patients with aHUS [75,76,78,79,82,83,84,85,86,87,88,89,90,91,92,93,94,95,96,97].

The efficacy and safety of eculizumab was also evaluated recently in 2 phase II prospective, multicenter, controlled clinical studies carried out in patients ≥12 years of age [98]. There were 17 and 20 enrolled patients resistant to plasma therapy and ongoing chronic plasma treatment, respectively. The authors reported that after 6 months of treatment with eculizumab, rates of hematological normalization (≥2 consecutive normal measurements of platelets and lactate dehydrogenase) reached 76% in resistant cases and 90% in chronic cases. Furthermore, patients without demonstrated mutations/antibodies responded well [98]. The authors stated that the improvement in renal function was maintained in extended studies (mean follow-up period of 62-64 weeks) [98].

Patient outcomes were reported as being poor among those treated with plasma therapy [17,49]. Moreover, the switch from plasma therapy to eculizumab has been shown to improve renal function even in patients with long-lasting and stable chronic kidney disease [99].

Based on these results, the FDA and the European Medicines Agency (EMA) approved in the United States and Europe, respectively, the indication for eculizumab in the treatment of aHUS. After having eculizumab as an important treatment option in aHUS patients, several guidelines were also published about the diagnosis and management of aHUS patients. The latest guideline was published by the American Society of Nephrology and early treatment with eculizumab was recommended as the first choice in the suspicion of aHUS in a pediatric patient. It was also highly recommended to use early eculizumab in any adult patient with suspected aHUS. In the case of unavailability of eculizumab, early and intensive plasmapheresis should be administered until eculizumab is an available option [57].

Today, clinical experience has clearly demonstrated that eculizumab is superior to plasma exchange for aHUS. Therefore, eculizumab should be considered the first line of therapy for aHUS when the diagnosis is reasonably unequivocal (e.g., recurrent cases, familial cases, posttransplant recurrence, idiopathic cases with TTP excluded). However, it takes a few weeks to get eculizumab due to logistic issues in Turkey, and it is recommended that plasmapheresis should be initiated as early as possible in any patient with symptoms of aHUS. Plasma exchange therapy is also initiated when TTP cannot be excluded based on the clinical and laboratory information available. Persistence of hemolysis or lack of improvement of renal function after 3-5 daily plasmapheresis treatments have to be regarded as the major criteria for uncontrolled TMA even if the platelet count has normalized and as an indication to switch the treatment to eculizumab [11,12,73].

It should be noted that eculizumab increases the patient’s susceptibility to certain serious infections, particularly meningococcal infections. To reduce the risk of infection, all patients with aHUS must also be vaccinated at least 2 weeks prior to receiving Soliris [100]. In emergency situations, until vaccination provides immunization, prophylactic antimeningococcal antibiotics should also be given during the first 2 weeks of the eculizumab treatment.

Eculizumab has changed the future perspectives of patients with aHUS and both the FDA and the EMA have approved it as life-long treatment. However, there are still some unresolved issues about follow-up, such as the optimal duration of eculizumab treatment and whether it can be stopped or how to stop the therapy. All of these questions can be resolved with data from large international prospective cohort studies.

**Conflict of Interest Statement**

The authors of this paper have no conflicts of interest, including specific financial interests, relationships, and/ or affiliations relevant to the subject matter or materials included.
